# The diversity of endophytic fungi in the above-ground tissue of two *Lycopodium* species in Poland

**DOI:** 10.1007/s13199-014-0291-1

**Published:** 2014-09-10

**Authors:** Julia Pawłowska, Mateusz Wilk, Anna Śliwińska-Wyrzychowska, Monika Mętrak, Marta Wrzosek

**Affiliations:** 1 Department of Systematics and Plant Geography, Faculty of Biology, The University of Warsaw, Al. Ujazdowskie 4, 00-478 Warsaw, Poland; 2 College of Inter-Faculty Individual Studies in Mathematics and Natural Sciences, The University of Warsaw, Żwirki i Wigury 93, 02-089 Warsaw, Poland; 3 Department of Botany and Plant Ecology, Institute of Chemistry, Environmental Protection and Biotechnology, Jan Długosz University in Częstochowa, Al. Armii Krajowej 13/15, 42-201 Częstochowa, Poland; 4 Department of Plant Ecology and Environmental Protection, The University of Warsaw, Al. Ujazdowskie 4, 00-478 Warsaw, Poland

**Keywords:** Lycophyte, Endophyte, Fungi, Diversity

## Abstract

Endophytes are a large and diverse group of fungi that colonize healthy plant tissues without causing any symptoms. The majority of studies have focused on angiosperm and conifer hosts and few have examined the endophytes of lycophytes. In the present study, we characterized culturable endophytic fungi in two closely related *Lycopodium* species (*L. annotinum* and *L. clavatum*) from pine, beech, oak and spruce forests across Poland. More than 400 strains were isolated but only 18 Ascomycete species were identified. Members of the Dothideomycetes dominated the fungal endophyte communities in *Lycopodium*. The most abundant taxa cultured were *Phoma brasiliensis* (from *L. clavatum*) and *Paraconiothyrium lycopodinum* (from *L. annotinum*). Five taxa were isolated exclusively from *L. annotinum*, but only two of them (*Paraconiothyrium lycopodinum* and *Mycosphaerella* sp.) were relatively abundant. Two taxa were only found in *L. clavatum*, namely: *Stagonospora pseudovitensis* and an unidentified Dothideomycete. The taxon assigned as Ascomycota 2 (SH219457.06FU) was isolated only from strobili of both host species. Direct PCR and cloning from *L. annotinum* shoots revealed a substantially greater endophyte richness compared with the results from culturing.

**Electronic supplementary material:**

The online version of this article (doi:10.1007/s13199-014-0291-1) contains supplementary material, which is available to authorized users.

## Introduction

*Lycopodium* (Lycopodiaceae, Lycopodiophyta, Plantae) is a genus of flowerless, vascular, terrestrial plants that reproduce sexually via homospores, and vegetatively from rhizomes. The Lycopod lineage diversified early and was the dominant vegetation in Carboniferous period (Ranker and Haufler 2008). Reports of fossils found in the Rhynie chert suggest that arbuscular mycorrhizal fungi and Lycopods co-evolved from the beginning (Remy et al. 1994; Taylor et al. 1999, 2004).

Endophytes form a large and phylogenetically diverse group of fungi that colonize healthy plant tissues without causing symptoms (Wilson 1995). Endophytes *sensu stricto* differ from mycorrhizal fungi by the fact that they reside entirely within plant tissues (Stone et al. 2004). Four classes of endophytes have been distinguished: 1) the clavicipitaceous endophytes, 2) the nonclavicipitaceous endophytes that colonize the whole plant, 3) the nonclavicipitaceous endophytes that colonize shoots, and 4) the nonclavicipitaceous endophytes that colonize roots (Rodriguez et al. 2009). All land plants studied to date, including lycophytes, are colonized by class three endophytes which are horizontally transmitted (Davis et al. 2003; Saikkonen et al. 1998). The representatives of this class occur in above-ground tissues, form localized infections and are remarkable for their high diversity even within individual hosts (e.g. Arnold 2007; Higgins et al. 2007). Many studies have tried to determine whether endophytes exhibit tissue- and host-specificity but the results are contradictory (Arnold et al. 2001; Higgins et al. 2007, 2011; Joshee et al. 2009; Moricca et al. 2012; Sun et al. 2012; Wearn et al. 2012), with indications of both host-specificity and host-generalism (see review of older reports by Zhou and Hyde (2001).

Only few studies have been devoted to examining the endophytic community of Lycopodiaceae (e.g. Budziszewska and Szypuła 2010; Chen et al. 2011; Wang et al. 2011), and two have been driven by an interest in identifying possible novel bioactive chemical compounds (e.g. Zhu et al. 2010; Xiang et al. 2013). Indeed, some endophytic fungi from lycophyte *Huperzia serrata* that is used in chinese medicine are known to produce bioactive metabolites such as huaspenones, (Xiang et al. 2013) or huperzine (see Zhu et al. 2010; Zhang et al. 2011, and literature therein). Only two studies, by Holm and Holm (1981) and by Engelhardt (1987), have been dedicated studying the fungal communities of above-ground organs of Lycopodiaceae.

Representatives of the Lycopodiaceae have been included in broader comparative studies of fungal endophytic communities in different plant groups (e.g. Higgins et al. 2007; U’Ren et al. 2012). Although the Lycopodiaceae have been studied for over 100 years, most researches have focused on the symbiotic relationships between the prothalli and mycorrhizal fungi (e.g. Bruchmann 1908; Freeberg 1962; Whittier 1977; Berch and Kendrick 1982; Schmid and Oberwinkler 1993; Read et al. 2000; Lee et al. 2001; Wang and Qiu 2006; Leake et al. 2008; Kessler et al. 2010a, b; Muthukumar and Prabha 2013). Even in this respect, only about 17 species of Lycopodiaceae have been studied (Wang and Qiu 2006). Non-mycorrhizal fungi (e.g. pathogens or saprotrophs) from Lycopodiaceae have also been examined in surveys (e.g. Hagen 1950; Holm and Holm 1984; Jaklitsch and Voglmayr 2012) or when preparing monographs of particular genera (e.g. *Massarina* by Aptroot 1998 or *Phaeosphaeria* by Leuchtmann 1984).

The aim of the present study was to characterize the fungal endophytic communities of two closely related *Lycopodium* species. We addressed the following questions: 1) How diverse are endophytic fungi from *Lycopodium* in temperate forest? 2) Is species community composition influenced by host plant, plant organ, geographic location of the host, or host habitat characteristics? 3) What is the taxonomic identity of hypothetical species isolated as endophytes?

## Materials and methods

### Host plant collection and localization of sampling sites

**Fig. 1:**
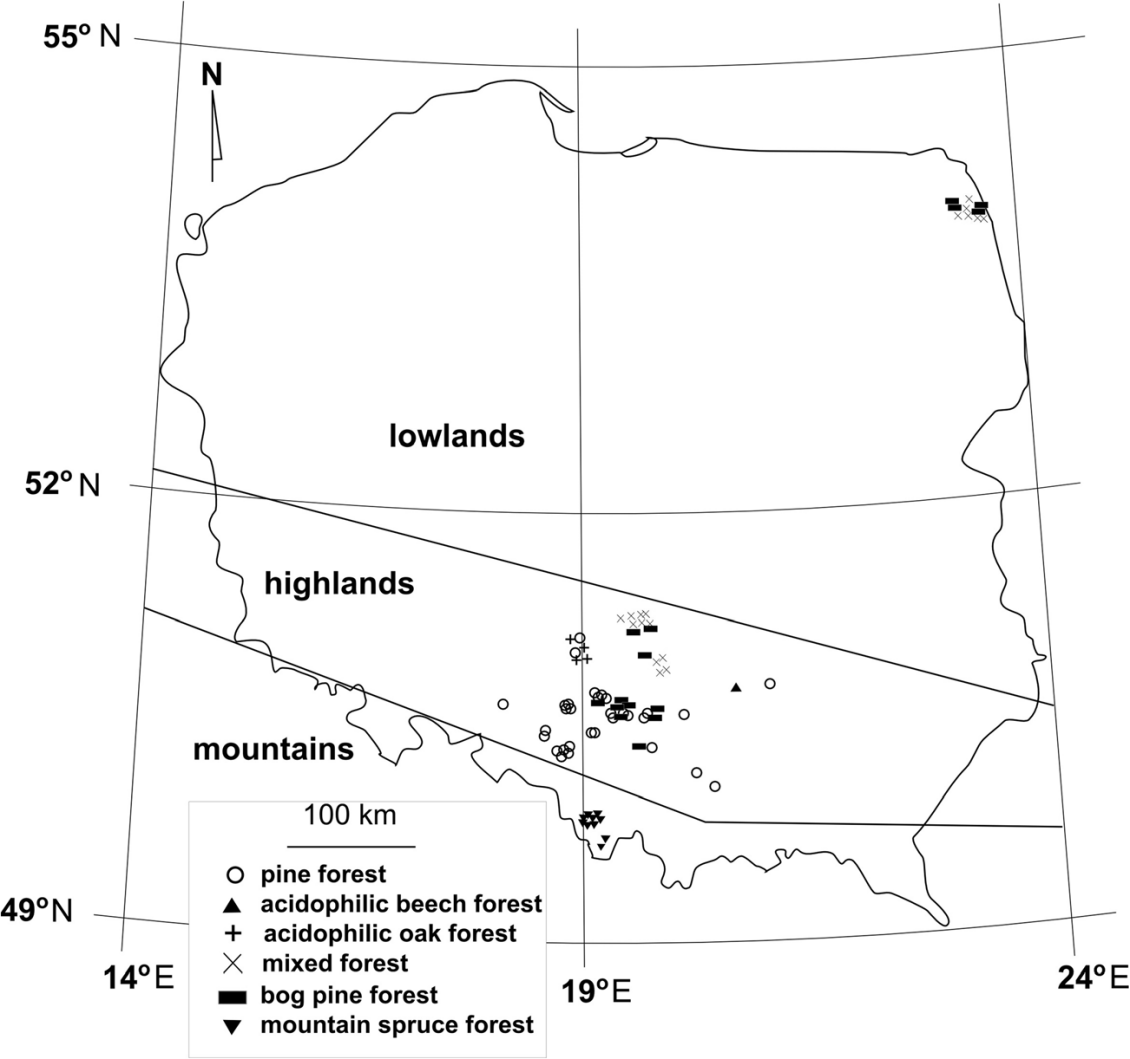
Distribution of sample sites

Shoots (8 individuals), strobili (15 individuals) and whole plants (shoots and strobili from the same individual; 13 individuals) of *L. annotinum* from 36 sites, and 10 shoots, 15 strobili, 2 whole plants (shoots and strobili from the same individual) of *L. clavatum* from 27 locations from temperate forests in Poland were collected (a total of 63 locations, 63 individuals and 78 samples; Fig. 1; see supplementary table S1). Sampling sites differed in terms of vegetation, with majority located in pine forests (27 sites); others in mixed forests (12 sites), acidophilic beech forests (1 site), acidophilic oak forests (4 sites), bog pine forests (11 sites), or mountain spruce forests (8 sites). Samples were further characterized by site elevation: lowlands (6 sites, 10 samples), highlands (49 sites, 58 samples), and mountains (8 sites, 10 samples; see supplementary table S1). Samples were collected in summer and autumn 2011 (see supplementary table S1). Each specimen was wrapped in a paper towel moistened with sterile water and placed in sterile plastic tube for transportation to the laboratory.

### Endophytes isolation and identification

Healthy shoots and strobili of *L. annotinum* and *L. clavatum* were washed in tap water and surface-sterilized by subsequent submersion of the plant tissue for 2 min in a 0.5 % sodium hypochlorite and for 2 min in 70 % ethanol. Finally, samples were rinse in sterile distilled water, as described by Davis et al. (2003). This method eliminates epiphytic bacteria, yeasts, and fast-growing Zygomycetes (Arnold et al. 2000). The absence of epiphytic fungi was then verified by imprinting sterilized plant fragments on control PDA plates (if no fungal growth was observed, the plant fragment was considered to have been effectively surface-sterilized). Subsequently, the samples of lycophytes were cut in 2 mm pieces. Eight segments per organ (shoot or strobili) per site were plated (624 segments in total) on 4 % potato dextrose agar (PDA; without antibiotics). All plates (control and samples) were incubated at room temperature (ca. 18^o^ C) for 10 weeks or until fungal growth was observed. Using aseptic technique, emergent hyphae were transferred and purified on sterile PDA plates. The endophytes were grouped into morphotypes as described by Wang et al. (2011). Identification of the isolated strains was done using Domsch et al. (1993), Samson et al. (2004), and Watanabe (2002). Ninety six morphotypes were distinguished.

Genomic DNA was extracted from pure cultures of a representative isolate of each fungal morphotype using the GeneMATRIX Plant & Fungi DNA Purification Kit (EURx Ltd., Gdańsk, Poland) following the manufacturer’s instructions. The complete ITS region was amplified using the primer pair ITS1f and ITS4 (White et al. 1990). PCR products were analyzed on a 1 % agarose gel stained with EtBr, and positive amplicons were purified using the GeneMATRIX Agarose-Out DNA Purification Kit (EURx Ltd). Purified DNA fragments were sequenced in both directions using the BigDye® Terminator v3.1 Cycle Sequencing Kit (Applied Biosystems, Carlsbad, CA, USA). Sequencing was performed at the Institute of Biochemistry and Biophysics, the Polish Academy of Sciences. Forward and reversed sequences were aligned into contigs and manually edited for errors using the BioEdit Sequence Alignment Editor v. 7.0.0 (Hall 1999).

ITS sequences of all (96) morphotypes were grouped into operational taxonomic units (OTUs) according to 98.5 % similarity using BioEdit Sequence Alignment Editor v. 7.0.0 (Hall 1999). This 98.5 % cutoff was selected and used for the hypothetical species assignment in the UNITE database (Kõljalg et al. 2013).

In order to estimate the fraction of unculturable endophytes, total genomic DNA was extracted (as described above) from sterilized *L. annotinum* shoot from site no. 30 (coordinates in supplement S1). The ITS and 5.8S rDNA regions were amplified using fungal specific primer pair ITS1-f and ITS4 (as described above). PCR products were ligated into pGEM-T Easy Vector (Promega, Leiden, The Netherlands) and cloned in *E. coli* JM109 competent cells (Promega) following the manufacturer’s instructions. Colony PCRs were performed using universal primer pair M13f and M13r.

Representative vouchers specimens for each OTU were deposited at the General Herbarium, University of Warsaw. Their numbers as well as the GenBank accession numbers of their sequences are presented in Table 1.

**Table 1:** Hypothetical species assignment of isolated strains based on massBLAST queries in UNITE database (for ITS sequences at 98.5 % of similarities). If the taxon assignment was done in different way than it is explained in footnotes. Isolates are presented in table according to their higher level classification (classes and orders)

Species hypotheses (SH) name	SH number	Voucher herbarium number	GenBank accession number	Taxon name used further in this paper
Dothideomycetes				
Capnodiales				
*Davidiella tassiana* (syn. *Mycosphaerella tassiana*)	SH196750.06FU	WA19047	JX981454	*Mycosphaerella tassiana*
*Mycosphaerella* sp.	SH195177.06FU	WA19033	JX981499	*Mycosphaerella* sp.
Dothideales				
*Aureobasidium pullulans*	SH206629.06FU	WA19043	JX981476	*Aureobasidium pullulans*
Pleosporales				
Ascomycota	SH224125.06FU	WA19030	JX629111	Ascomycota 1
Dothideomycetes	SH196053.06FU	WA19143	JX981466	Dothideomycetes
*Phoma brasiliensis*	SH202145.06FU	WA19053	JX981489	*Phoma brasiliensis*
Pleosporales	SH233951.06FU	WA19052	JX981474	Pleosporales
*Pyrenophora chaetomioides*	SH227032.06FU	WA19141	JX981468	*Pyrenophora chaetomioides*
*Stagonospora pseudovitensis*	SH199974.06FU	WA19138	JX981472	*Stagonospora pseudovitensis*
no SH proposed^a^		WA19040	JX981452	*Alternaria* sp.^a^
no SH proposed^b^		WA19015	JX629096	*Paraconiothyrium lycopodinum* ^b^
no SH proposed^c^		WA19023	JX629104	*Paraconiothyrium polonense* ^c^
no SH proposed^d^		WA19031	JX629112	*Paraphaeosphaeria* sp.^d^
Leotiomycetes				
Helotiales				
Helotiales	SH232201.06FU	WA19148	JX981467	Helotiales
*Phacidium lacerum*	SH108595.06FU	WA19039	JX981469	*Phacidium lacerum*
Sordariomycetes				
Hypocreales				
Ascomycota	SH219457.06FU	WA19121	JX981457	Ascomycota 2
*Trichoderma viride* (syn. *Hypocrea rufa*)	SH222750.06FU	WA19150	JX981461	*Trichoderma viride*
Xylariales				
*Leiosphaerella lycopodina*	SH230356.06FU	WA19125	JX981475	*Leiosphaerella lycopodina*

^a^The closest sequences in blast queries are*: Alternaria tenuissima* (KJ082100; 100 % similarity) and *Alternaria alternata* (KJ082099; 100 % similarity) thus the strain is identified as *Alternaria* sp.

^b^The strain was identified by authors based on morphology and was described recently as new species *Paraconiothyrium lycopodinum* (Sacc. & Paol.) J. Pawłowska, Wilk, Śliwińska-Wyrzychowska, Mętrak & Wrzosek (Crous et al. 2013)

^c^The strain was identified by authors based on morphology and was described recently as new species *Paraconiothyrium polonense* J. Pawłowska, Wilk, Śliwińska-Wyrzychowska, Mętrak & Wrzosek (Crous et al. 2013)

^d^The closest sequences in blast queries are*: Paraphaeosphaeria sporulosa* (JX629112; 100 % similarity) and *Paraphaeosphaeria neglecta* (JX496204; 100 % similarity) thus the species is identified as *Paraphaeosphaeria* sp.

### Data analysis

ITS sequences representing each morphotype were queried against the UNITE database using the massBLAST algorithm (http://unite.ut.ee; Abarenkov et al. 2010; Kõjalg et al. 2005). Species Hypothesis (SH) at 98.5 % of sequence similarity were used to identify isolated morphotypes (Kõljalg et al. 2013). If SH at 98.5 % of similarity was not proposed, the results of BLASTn (Altschul et al. 1997) searches were used to estimate taxonomic position of isolate and then detailed explanation concerning each case were given in Table 1.

The colonization factor (CF%; called also isolation frequency or colonization frequency) was calculated as the total number of plant segments colonized by fungi divided by the total number of all incubated segments, expressed as its percentage (Hata and Futai 1995). Relative abundance was calculated as the number of all isolates of a given taxon divided by the total number of isolates of all taxa. Frequency was calculated as the number of host individuals of fungal taxon isolated divided by the total number of all host individuals (Sun et al. 2012). Species diversity was evaluated using Shannon’s Diversity Index (Shannon 1948) and Fisher’s alpha (Fisher et al. 1943). The species evenness was estimated with Pielou’s evenness index (Pielou 1966). The similarity of endophytic communities between different sampling sites (63) was evaluated using the Jaccard similarity coefficient (Jaccard 1912). Sample-based species accumulation curves for different hosts and organs were calculated and estimations of total richness were compared using the Jackknife 1 extrapolation algorithm. To correct for unequal sample sizes, the data was reduced to the smallest, consistent sample size (*N* = 12, for shoots of *Lycopodium clavatum*). All indices were calculated using EstimateS v.9.1.0. (Colwell 2006).

Statistical analyses were then performed in four different variants in order to compare the influence of host plant, type of plant organ, host geographical location, and host habitat characteristics: (1) comparison between *Lycopodium calavatum* and *Lycopodium annotinum*; (2) comparison between shoots and strobili; (3) comparison between the samples coming from lowlands, highlands and mountains; (4) comparison between the samples collected from mixed pine, fresh pine, acidophilic oak, mountain spruce, pine bog, and acidophilic beech forests. To correct for inconsistent sample sizes in diversity and richness comparisons between samples, the data was reduced to the smallest sample size (randomly selected host individuals) as proposed by Davis and Shaw (2008).

In all the described variants, after the performance of the Shapiro-Wilk tests for normality for each variant separately, Kruskal-Wallis one-way analysis of variance was chosen as an appropriate method of comparison (non-parametric test for groups of unequal size) (Kruskal and Wallis 1952).

Variation in community composition was examined as a function of host, organ, location and vegetation type using three different ecological community similarity measures: Jaccard’s similarity index, the Bray-Curtis coefficient and Euclidean distance. Resulting matrices were represented using non-metric multidimensional scale (NMDS) plots. To avoid pseudoreplication, data from different tissues types of the same host individual were pooled for NMDS analyses that examine the effect of host, location, and vegetation type. The Kruskal’s stress was used to decide which grouping of data is the most accurate (commonly acceptable when lower than 0.2) (Ek-Ramos et al. 2013).

Additionally, the correlation between Jaccard similarity coefficient and geographic distance was tested for significance using simple Mantel test. The geographical distances (in km) based on coordinates of collection sites (WGS84 system) were determined using own program written in C (assuming that the earth is a perfect sphere and its radius is 6,378 km).

All statistical analyses were calculated using STATISTICA v. 10 and PAST v. 2.16 software (Hammer et al. 2001).

## Results

**Fig. 2:**
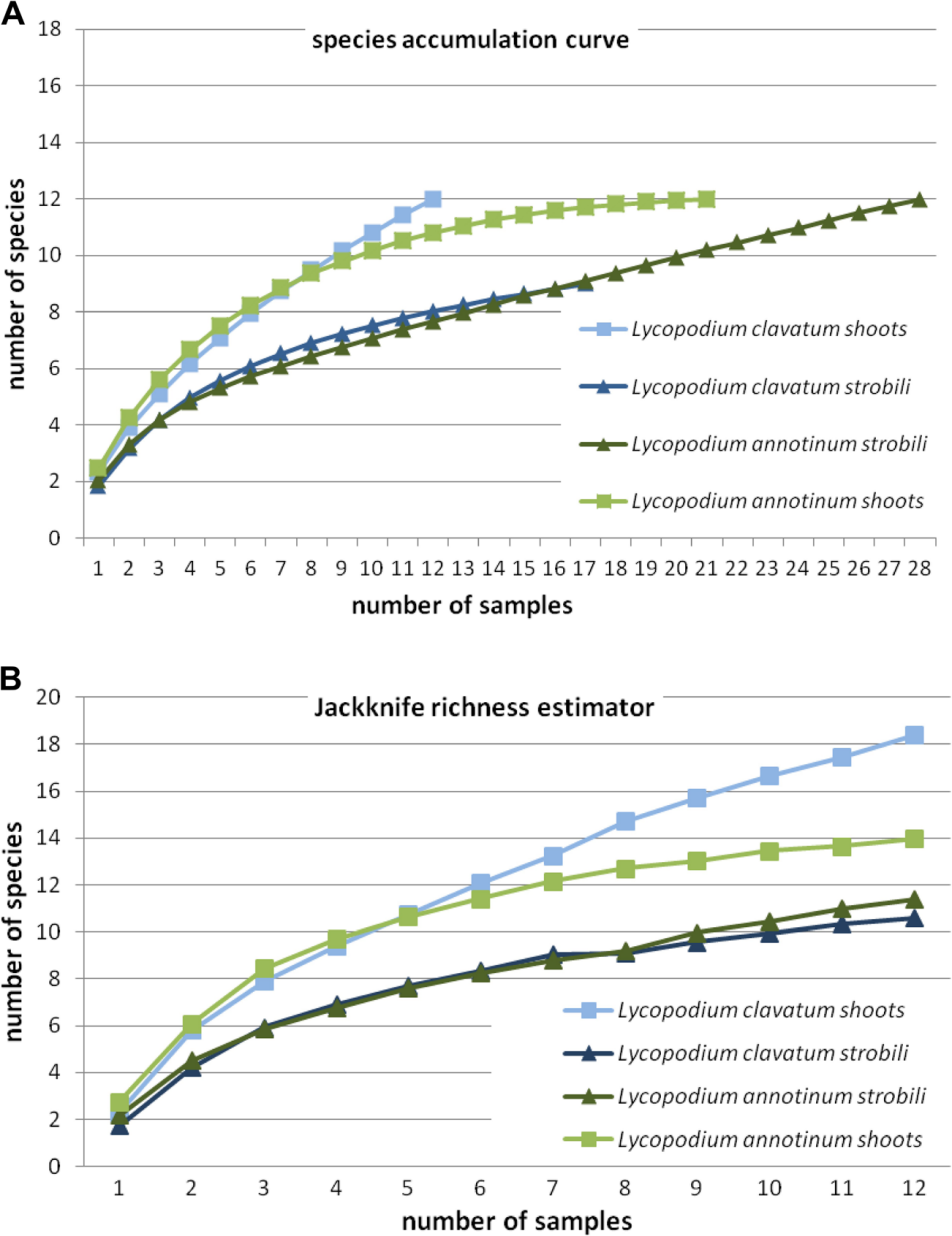
**a** Species accumulation curve for analyzed host species and organs. **b** Jackknife 1 total richness estimator curve. Sample sizes were standardized to the smallest consistent size (*N* = 12, from shoots of *Lycopodium clavatum*)

**Fig. 3:**
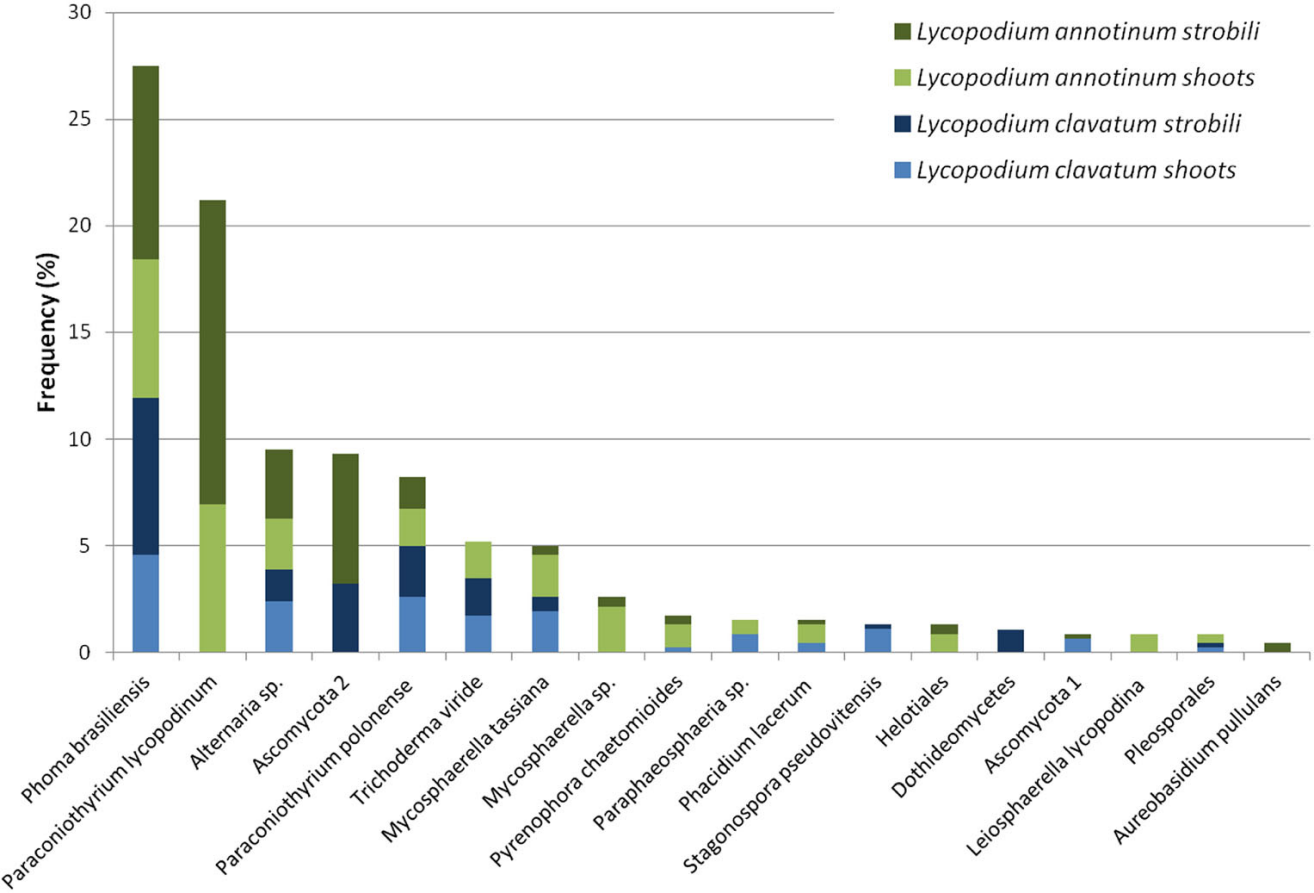
Frequency (%; calculated for total of 78 individuals)’of the endophytic fungal isolates in shoots and strobili of the *Lycopodium* species

From 624 *Lycopodium* segments (representing a total of 63 locations, 63 individuals and 78 samples), a total of 458 isolates, representing 96 morphotypes, 18 OTUs (hypothetical species defined in UNITE database) (Table 1), which belong only to the Ascomycota*,* were isolated during this study. Representatives of Dothideomycetes were isolated the most frequently. However, only in the case of *L. annotinum* shoots did the accumulation curve reach an asymptote. The Jacknife richness estimator did not reach an asymptote which indicates that richness would continue to increase with further sampling (Fig. 2). The most abundant taxa were *Phoma brasiliense* in *L. clavatum* and *Paraconiothyrium lycopodinum* in *L. annotinum* (Table 2). The highest frequency was observed also for these two taxa (Fig. 3)*.*

**Table 2:** Relative abundance (%) of endophytic fungi isolated from shoots and strobili of two *Lycopodium* species (the most abundant taxa for each host are shown in bold)

	*Lycopodium clavatum*			*Lycopodium annotinum*		
	Shoots	Strobili	Total	Shoots	Strobili	Total
*Alternaria* sp.	2.38	1.52	3.90	2.38	3.25	5.63
Ascomycota 1	0.65	0.00	0.65	0.00	0.22	0.22
Ascomycota 2	0.00	3.25	3.25	0.00	6.06	6.06
*Aureobasidium pullulans*	0.00	0.00	0.00	0.00	0.43	0.43
Dothideomycetes	0.00	1.08	1.08	0.00	0.00	0.00
Helotiales	0.00	0.00	0.00	0.87	0.43	1.30
*Leiosphaerella lycopodina*	0.00	0.00	0.00	0.87	0.00	0.87
*Mycosphaerella* sp.	0.00	0.00	0.00	2.16	0.43	2.60
*Mycosphaerella tassiana*	1.95	0.65	2.60	1.95	0.43	2.38
***Paraconiothyrium lycopodinum***	0.00	0.00	0.00	**6.93**	**14.29**	**21.21**
*Paraconiothyrium polonense*	2.60	2.38	4.98	1.73	1.52	3.25
*Paraphaeosphaeria* sp.	0.87	0.00	0.87	0.65	0.00	0.65
*Phacidium lacerum*	0.43	0.00	0.43	0.87	0.22	1.08
***Phoma brasiliensis***	**4.55**	**7.36**	**11.9**	6.49	9.09	15.58
Pleosporales	0.22	0.22	0.43	0.43	0.00	0.43
*Pyrenophora chaetomioides*	0.22	0.00	0.22	1.08	0.43	1.52
*Stagonospora pseudovitensis*	1.09	0.22	1.31	0.00	0.00	0.00
*Trichoderma viride*	1.73	1.73	3.46	1.73	0.00	1.73

None of the isolated taxa was present in all samples. There were 5 taxa isolated exclusively from *L. annotinum*, but only two of them (*Paraconiothyrium lycopodinum* and *Mycosphaerella* sp.) were relatively abundant (more than 5 isolates). There were also two taxa that were exclusive for *L. clavatum*, namely: *Stagonospora pseudovitensis* and unidentified Dothideomycetes (SH196053.06FU). The taxon assigned as Ascomycota 2 (SH219457.06FU) was isolated only from strobili of both host species.

Although we analyzed many more segments of *L. annotinum* than *L. clavatum*, the number of isolated species as well as colonization factor and values of species diversity indices for random 12 samples (the smallest sample size) were very similar (Table 3).

**Table 3:** Overall colonization factor (%), species richness, Shannon’s diversity index, Fisher’s alpha values and Pielou’s evenness index of endophytic fungi isolated from shoots and strobili of two *Lycopodium* species

	*Lycopodium clavatum*			*Lycopodium annotinum*			
	Strobili		Shoots	Strobili		Shoots	
	Values for all samples	Values for 12 random individuals (the smallest sample size)	Values for all (12) samples	Values for all samples	Values for 12 random individuals (the smallest sample size)	Values for all samples	Values for 12 random individuals (the smallest sample size)
Number of host individuals	17	12	12	28	12	21	12
Number of segments	136	96	96	224	96	168	96
Total number of isolates	85	60	77	167	72	129	74
Colonization factor (%)	62.5	62.5	80.2	74.5	74.5	76.8	76.8
Total number of species	9	8	12	12	8	12	11
Shanon’s diversity index	1.75	1.24	2.1	1.67	0.72	2.14	1.22
Fisher’s alpha	2.54	1.79	3.98	2.96	1.27	3.23	1.85
Pielou’s evenness index	0.796	0.596	0.845	0.672	0.803	0.861	0.892

There were no significant differences in total number of fungal species (*p* = 0.1872), total number of isolates (*p* = 0.1455), colonization factor (*p* = 0.1455), Shannon diversity (*p* = 0.2263), Fisher’s alpha (*p* = 0.0953) and evenness (*p* = 0.2206) between the two *Lycopodium* species.

The total number of fungal species (*p* = 0.1623), total number of isolates (*p* = 0.8355), colonization factor (*p* = 0.8355), Shannon diversity (*p* = 0.1073), Fisher’s alpha (*p* = 0.8020) and evenness (*p* = 0.1305) between different site locations (mountain, highland, lowland) and vegetation type of sample site (mixed, pine, oak, mountain spruce, pine bog, or beech forests) (p values: 0.2438, 0.1183, 0.1183, 0.1947, 0.5178, 0.6572 respectively) also did not statistically differ.

Only the Shannon diversity index (*p* = 0.0294) was significantly higher in shoots than in strobili for both *Lycopodium* species (see also Table 3). This pattern is also visible in species accumulation and in Jacknife 1 richness estimator curves (Fig. 2).

**Fig. 4:**
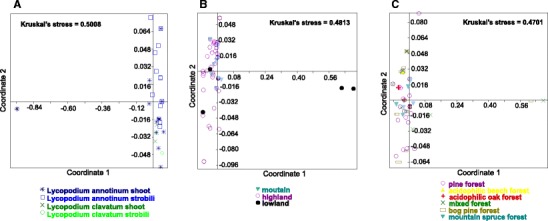
Non-metric multidimensional scale plots of Jaccard’s similarity indexes representing effect of host, organ, location and vegetation type on fungal endophytic communities of *Lycopodium annotinum* and *Lycopodium clavatum*. Each point represents a single endophyte community from a particular individual (**a** for 15 whole plants, **b** and **c** for 63 individual each from different site). Kruskal’s stress values are indicated

Cluster analysis of endophytic community similarity measures presented in two-dimensional NMDS plots revealed that these communities are neither lycophyte-host-related , nor organ-related (even though differences is diversity between organs are significant). Variation in endophytic community structure could neither be explained by site vegetation type nor by geographic region of host plant origin (Fig. 4). The use of different ecological similarity measures did not significantly affect the observed patterns (data not shown). In most cases Kruskal’s stress values were too high (>0.2) to confidently discern any pattern. However, the two samples of *L. annotinum* from the lowland that had a very different endophytic communy from samples collected from other elevations.

The shoots of *L. annotinum* from site 30 (as described in supplement S1) on PDA medium yielded only two species *Leiosphaerella lycopodina* and *Mycosphaerella* sp., while in the cloning experiment with the plant tissue, seven different sequences were obtained (Table 4). The sequences of both species isolated in culture were also present in cloned sequences and they were the most frequent ones. However, they still constituted less than a half of all obtained sequences.

**Table 4:** Comparison of SH proposed by UNITE for sequences that were received in culture based approach with ones from cloning experiment (from *L. annotinum* sample from site 30 in Puszcza Augustowska)

SH name proposed in UNITE for the sequences received in culture based approach (SH number)	number of isolates	SH name proposed in UNITE for the sequences received in cloning based approach (SH number)	GB number of closest sequence	% of similarity	% of clones
*Leiosphaerella lycopodina* (SH230356.06FU)	2	*Leiosphaerella lycopodina* (SH230356.06FU)	JF440975	99 %	22 %
*Mycosphaerella* sp. (SH195177.06FU)	4	*Mycosphaerella* sp. (SH195177.06FU)	EU167581	94 %	22 %
		*Pseudocercosporella* sp. (SH195098.06FU)	AY805600	100 %	6 %
		*Botryosphaeria corticis* (SH206853.06FU)	HQ529751	99 %	21 %
		Tubeufiaceae (SH230727.06FU)	AY916453	99 %	6 %
		*Chalara dualis* (SH208308.06FU)	EF029209	98 %	17 %
		*Exobasidium japonicum* (SH204828.06FU)	EU692772	96 %	6 %

## Discussion

Higgins et al. (2007) found a low diversity of endophytes in *Huperzia* (Fisher’s alpha 1.27–3.98) while U’Ren et al. (2012) found a very high diversity of endophytes in this genus in samples from Alaska (Fisher’s alpha 15.64). These studies both used a culture-based approach for characterization of the fungal endophyte communities. This likely resulted in a serious underestimation of the fungal diversity. This is supported by our species accumulation curve (Fig. 2) as well as by the results of our cloning trial, where only less than half of all endophytic fungi present in the sample appeared in culture (Table 4). Another possibility is that species diversity may vary with environmental factors at sample sites but more study is required.

Among the isolated taxa, five were found exclusively in *L. annotinum*, of which only two (*Paraconiothyrium lycopodinum* and *Mycosphaerella* sp.) were abundant (more than 5 isolates). The isolate Ascomycota 2 (SH219457.06FU) was restricted to the strobili, regardless of the host species which suggests some level of tissue-preference, for this isolate. However, this might be a result of isolate rarity.

Cluster analysis of endophytic community similarity measures presented in two-dimensional NMDS plots revealed that only two samples from *L. annotinum* from lowland have very different endophytic community than all others. This pattern could be explained by the relatively high abundance of *Mycosphaerella* sp. and by the fact that both sites are relatively close together (240 m apart). As lycophytes can reproduce asexually, it is possible that the plants represent the same host individual. The abundant presence of *Mycosphaerella* sp. at these sites should be verified in further research.

Some of taxa isolated in our study are common and widespread saprotrophic fungi, e.g. *Hypocrea rufa* and its anamorph *Trichoderma viride* but they have been isolated as endophytes previously (Jaklitsch et al. 2006), but not from lycophytes. Among other taxa we isolated are well known plant pathogens, e.g. *Leiosphaerella lycopodina* that has been recorded from *Lycopodium annotinum* several times (e.g. Jaklitsch and Voglmayr 2012) but never described to date as an endophyte.

One of the most abundant taxon in our study was *Paraconiothyrium lycopodinum* that could be identified as *Coniothyrium lycopodinum* Sacc. & Paol. that was previously isolated as a possible pathogen from *L. annotinum* (Saccardo 1889). These two taxa were recently synonimized by Pawłowska et al. (in Crous et al. 2013) as *Paraconiothyrium lycopodinum* (Sacc. & Paol.) J. Pawłowska, Wilk, Śliwińska-Wyrzychowska, Mętrak & Wrzosek, *comb. nov*. In spite of the fact that several *Paraconiothyrium* species have been isolated as endophytes from asymptomatic photosynthetic tissues of plant species (e.g. Abreu et al. 2010), including lycophytes (Budziszewska et al. 2011; Wang et al. 2011), in general they are still regarded as plant pathogens (Damm et al. 2008).

It is interesting that some fungi isolated in our study are known to be, or bear close affinities, to plant pathogens. This is a common finding of studies on endophytic fungal communities (e.g. Moricca et al. 2012; Sun et al. 2012; Wearn et al. 2012 and references therein). This has led to the hypothesis that endophytism is a common phase in the life cycle of many fungal pathogens (e.g. Carroll 1988; Joshee et al. 2009; Rodriguez and Redman 1997; Schulz and Boyle 2005). Recently, Delaye et al. (2013) performed an analysis of the evolutionary stability of biotropic, necrotrophic, and endophytic lifestyles of 163 fungal strains. They suggest that while biotrophy appears to be a stable trait, asymptomatic endophytes can easily switch to necrotrophy, even at an ecological timescale. Not only is this of paramount importance for understanding the mechanisms and factors underlying alterations in fungal lifestyle, but it is also significant for studies on endophytic fungal communities. A large number of fungal species described in 19th century that often have no holotypes and are in desperate need for revision using molecular approaches. These fungi are still found on lists of fungal taxa recorded from a particular plant hosts (e.g. Farr and Rossman 2012). They were regarded as pathogens or saprotrophs, partially because molecular identification of endophytes was beyond the technical abilities at the time. Current investigations of endophytic fungal communities often exclusively apply molecular methods (Rajala et al. 2013). However a combination of these methods with culturing is best (e.g. Arnold and Lutzoni 2007). A study of the endophytic community based on molecular identification and culturing with direct observation of fungal communities on the plant material, has been successfully applied by Chaverri and Gazis (2011) and can shed more light on the question of the ‘endophytic continuum’. In this way it may also be possible to link of fungal taxa described in inventories like those by Engelhardt (1987) and by Holm and Holm (1981) with sequences of unidentified endophytes generated in numerous molecular studies.

Finally, the possible roles of these endophytic fungi in interactions with lycophytes are unknown. To date, no research has been performed on whether such endophytes may in any way enhance lycophyte fitness, make them more competitive within their habitats, deter potential animal herbivores or protect from pathogenic fungi or bacteria. There is clearly much more research to be done.

## Electronic supplementary material

Below is the link to the electronic supplementary material.

Table S1 (DOCX 25 kb)

## References

[CR1] Abarenkov K, Nilsson RH, Larsson KH, Alexander IJ, Eberhardt U, Erland S, Høiland K, Kjøller R, Larsson E, Pennanen T, Sen R, Taylor AFS, Tedersoo L, Ursing BM, Vrålstad T, Liimatainen K, Peintner U, Kõljalg U (2010). The UNITE database for molecular identification of fungi—recent updates and future perspectives. New Phytol.

[CR2] Abreu LM, Almeida AR, Salgado M, Pfenning LH (2010). Fungal endophytes associated with the mistletoe *Phoradendron perrottettii* and its host tree *Tapirira guianensis*. Mycol Prog.

[CR3] Altschul SF, Madden TL, Schaffer AA, Zhang J, Zhang Z, Miller W, Lipman DJ (1997). Gapped BLAST and PSI-BLAST: a new generation of protein database search programs. Nucleic Acids Res.

[CR4] Aptroot A (1998). A world revision of *Massarina* (Ascomycota). Nova Hedwigia.

[CR5] Arnold AE (2007). Understanding the diversity of foliar fungal endophytes: progress, challenges, and frontiers. Fungal Biol Rev.

[CR6] Arnold AE, Maynard Z, Gilbert GS (2001). Fungal endophytes in dicotyledonous neotropical trees: patterns of abundance and diversity. Mycol Res.

[CR7] Arnold AE, Maynard Z, Gilbert GS, Coley PD, Kursar TA (2000). Are tropical fungal endophytes hyperdiverse?. Ecol Lett.

[CR8] Arnold AE, Lutzoni F (2007). Diversity and host range of foliar fungal endophytes: are tropical leaves biodiversity hotspots?. Ecology.

[CR9] Berch SM, Kendrick B (1982). Vesicular-arbuscular mycorrhizae of Southern Ontario ferns and fern-allies. Mycologia.

[CR10] Bruchmann H (1908). Das Prothallium von *Lycopodium complanatum* L. Bot Zeit.

[CR11] Budziszewska J, Szypuła W (2010). Influence of site conditions on the diversity of endophytic fungi of clubmoss species *Huperzia selago* (L.) Bernh. ex Schrank et Mart. Pol J Ecol.

[CR12] Budziszewska J, Szypuła W, Wilk M, Wrzosek M (2011). *Paraconiothyrium babiogorense* sp. nov., a new endophyte from fir club moss *Huperzia selago* (Huperziaceae). Mycotaxon.

[CR13] Carroll GC (1988). Fungal endophytes in stems and leaves: from latent pathogen to mutualistic symbiont. Ecology.

[CR14] Chaverri P, Gazis RO (2011). Linking ex planta fungi with their endophytic stages: *Perisporiopsis*, a common leaf litter and soil fungus, is a frequent endophyte of *Hevea* spp. and other plants. Fung Ecol.

[CR15] Chen XY, Qi YD, Wei JH, Zhang Z, Wang DL, Feng JD, Gan BC (2011). Molecular identification of endophytic fungi from medicinal plant *Huperzia serrata* based on rDNA ITS analysis. World J Microbiol Biotechnol.

[CR16] Colwell RK (2006) EstimateS: Statistical estimation of species richness and shared species from samples. Version 8. User’s Guide and application published at: http://purl.oclc.org/estimates. Department of Ecology and Evolutionary Biology, University of Connecticut, Storrs, USA

[CR17] Crous PW, Wingfield MJ, Guarro J, Cheewangkoon R, van der Bank M, Swart WJ, Stchigel AM, Cano-Lira JF, Roux J, Madrid H, Damm U, Wood AR, Shuttleworth LA, Hodges CS, Munster M, de Jesús Yáñez-Morales M, Zúñiga-Estrada L, Cruywagen EM, de Hoog GS, Silvera C, Najafzadeh J, Davison EM, Davison PJN, Barrett MD, Barrett RL, Manamgoda DS, Minnis AM, Kleczewski NM, Flory SL, Castlebury LA, Clay K, Hyde KD, Maússe-Sitoe SND, Chen S, Lechat C, Hairaud M, Lesage-Meessen L, Pawlowska J, Wilk M, Sliwinska-Wyrzychowska A, Metrak M, Wrzosek M, Pavlic-Zupanc D, Maleme HM, Slippers B, Mac Cormack WP, Archuby DI, Grünwald NJ, Tellería MT, Dueñas M, Martín MP, Marincowitz S, de Beer ZW, Perez CA, Gené J, Marin-Felix Y, Groenewald JZ (2013). Fungal planet description sheets: 154–213. Persoonia.

[CR18] Delaye L, Garcia-Guzman G, Heil M (2013). Endophytes versus biotrophic and necrotrophic pathogens—are fungal lifestyles evolutionarily stable traits?. Fungal Divers.

[CR19] Damm U, Verkley GJM, Crous PW, Fourie PH, Haegi A, Riccioni L (2008). Novel *Paraconiothyrium* species on stone fruit trees and other woody hosts. Persoonia.

[CR20] Davis EC, Franklin JB, Shaw AJ, Vilgalys R (2003). Endophytic *Xylaria* (*Xylariaceae*) among liverworts and angiosperms: phylogenetics, distribution, and symbiosis. Am J Bot.

[CR21] Davis EC, Shaw AJ (2008). Biogeographic and phylogenetic patterns in diversity of liverwort-associated endophytes. Am J Bot.

[CR22] Domsch KH, Gams W, Anderson TH (1993) Compendium of soil fungi. Vol. I & II; IHW—Verlag. Eching, Germany, p 859 and p. 405

[CR23] Ek-Ramos MJ, Zhou W, Valencia CU, Antwi JB, Kalns LL, Morgan GD, Kerns DL, Sword GA (2013). Spatial and temporal variation in fungal endophyte communities isolated from cultivated cotton (*Gossypium hirsutum*). PLoS One.

[CR24] Engelhardt K (1987) Perfekte Ascomyceten an zwei Bärlapparten des Lichtenfelser Forstes. In: Band A (ed) Pilzflora Nordwestoberfrankens. Weidhausen bei Coburg 11, pp 33–38

[CR25] Farr DF, Rossman AY (2012) Fungal Databases, Systematic Mycology and Microbiology Laboratory, ARS, USDA. http://nt.ars-grin.gov/fungaldatabases/; Accessed 4 Sept 2013

[CR26] Fisher RA, Corbet AS, Williams CB (1943). The relation between the number of species and the number of individuals in a random sample of an animal population. J Anim Ecol.

[CR27] Freeberg JA (1962). *Lycopodium* prothalli and their endophytic fungi as studied in vitro. Am J Bot.

[CR28] Hagen A (1950). Notes on Arctic fungi. II. Fungi collected by Dr P. F. Scholander on the Swedisch-Norwegian Arctic expedition 1931. Nor Polarinst Skr.

[CR29] Hall TA (1999). BioEdit: a user-friendly biological sequence alignment editor and analysis program for Windows 95/98/NT. Nucl Acids Symp Ser.

[CR30] Hammer Ř, Harper DAT, Ryan PD (2001). PAST: paleontological statistics software package for education and data analysis. Palaeontol Electron.

[CR31] Hata K, Futai K (1995). Endophytic fungi associated with healthy pine needles and needles infested by the pine needle gall midge, *Thecodiplosis japonensis*. Can J Bot.

[CR32] Higgins LK, Arnold AE, Miadlikowska J, Sarvate SD, Lutzoni F (2007). Phylogenetic relationships, host affinity, and geographic structure of boreal and arctic endophytes from three major plant lineages. Mol Phylogenet Evol.

[CR33] Higgins HL, Coley PD, Kursar TA, Arnold AE (2011). Culturing and direct PCR suggest prevalent host generalism among diverse fungal endophytes of tropical forest grasses. Mycologia.

[CR34] Holm K, Holm L (1984). A contribution to the mycoflora of Iceland. Acta Bot Isl.

[CR35] Holm L, Holm K (1981). Ascomycetes on Nordic Lycopods. Karstenia.

[CR36] Jaccard P (1912). The distribution of the flora in the alpine zone. New Phytol.

[CR37] Jaklitsch WM, Samuels GJ, Dodd SL, Lu B-S, Druzhinina IS (2006). *Hypocrea rufa/Trichoderma viride*: a reassessment, and description of five closely related species with and without warted conidia. Stud Mycol.

[CR38] Jaklitsch WM, Voglmayr H (2012). Phylogenetic relationships of five genera of Xylariales and *Rosasphaeria* gen. nov. (Hypocreales). Fungal Divers.

[CR39] Joshee S, Paulus BC, Park D, Johnston PR (2009). Diversity and distribution of fungal foliar endophytes in New Zealand *Podocarpaceae*. Mycol Res.

[CR40] Kessler M, Jonas R, Cicuzza D, Kluge J, Piątek K, Naks P, Lehnert M (2010). A survey of the mycorrhization of Southeast Asian ferns and lycophytes. Plant Biol.

[CR41] Kessler M, Jonas R, Strasberg D, Lehnert M (2010). Mycorrhizal colonizations of ferns and lycophytes on the island of La Reunion in relation to nutrient availability. Basic Appl Ecol.

[CR42] Kõjalg U, Larsson K-H, Abarenkov K, Nilsson RH, Alexander IJ, Eberhardt U, Erland S, Høiland K, Kjøller R, Larsson E, Pennanen T, Sen R, Taylor AFS, Tedersoo L, Vrålstad T, Ursing BM (2005). UNITE: a database providing web-based methods for the molecular identification of ectomycorrhizal fungi. New Phytol.

[CR43] Kõljalg U, Nilsson RH, Abarenkov K, Tedersoo L, Taylor AFS, Bahram M, Bates ST, Bruns TD, Bengtsson-Palme J, Callaghan TM, Douglas B, Drenkhan T, Eberhardt U, Dueñas M, Grebenc T, Griffith GW, Hartmann M, Kirk PM, Kohout P, Larsson E, Lindahl BD, Lücking R, Martín MP, Matheny PB, Nguyen NH, Niskanen T, Oja J, Peay KG, Peintner U, Peterson M, Põldmaa K, Saag L, Saar I, Schüßler A, Scott JA, Senés C, Smith ME, Suija A, Taylor DL, Telleria MT, Weiß M, Larsson K-H (2013). Towards a unified paradigm for sequence-based identification of fungi. Mol Ecol.

[CR44] Kruskal W, Wallis WA (1952). Use of ranks in one-criterion variance analysis. J Am Stat Assoc.

[CR45] Leake JR, Cameron DD, Beerling DJ (2008). Fungal fidelity in the mycoheterotroph-to-autotroph life cycle of Lycopodiaceae: a case of parental nurture?. New Phytol.

[CR46] Lee J-K, Eom A-H, Lee S-S, Lee CH (2001). Mycorrhizal symbioses found in roots of fern and its relatives in Korea. J Plant Biol.

[CR47] Leuchtmann A (1984). Über Phaeosphaeria Miyake und andere bitunicate Ascomyceten mit mehrfach querseptierten Ascosporen. Sydowia.

[CR48] Moricca S, Ginetti B, Ragazzi A (2012). Species- and organ-specificity in endophytes colonizing healthy and declining Mediterranean oaks. Phytopatologia Mediterr.

[CR49] Muthukumar T, Prabha K (2013). Arbuscular mycorrhizal and septate endophyte fungal associations in lycophytes and ferns of south India. Symbiosis.

[CR50] Pielou EC (1966). The measurement of diversity in different types of biological collections. J Theor Biol.

[CR51] Rajala T, Velmala SM, Tuomivirta T, Haapanen M, Müller M, Pennanen T (2013). Endophyte communities vary in the needles of Norway spruce clones. Fungal Biol.

[CR52] Ranker TA, Haufler CH (2008). Biology and evolution of Ferns and Lycophytes.

[CR53] Read DJ, Duckett JG, Francis R, Ligrone R, Russell A (2000). Symbiotic fungal associations in ‘lower’ plants. Phil Trans R Soc Lond B.

[CR54] Remy W, Taylor TN, Hass H, Kerp H (1994). Four hundred-million-year-old vesicular arbuscular mycorrhizae. Proc Natl Acad Sci U S A.

[CR55] Rodriguez RJ, Redman RS (1997). Fungal life-styles and ecosystem dynamics: biological aspects of plant pathogens, plant endophytes and saprophytes. Adv Bot Res.

[CR56] Rodriguez RJ, White JF, Arnold AE, Redman RS (2009). Fungal endophytes: diversity and functional roles. New Phytol.

[CR57] Saccardo PA (1889). Mycetes Sibirici. Bull Soc R Bot Belg.

[CR58] Saikkonen K, Faeth SH, Helander M, Sullivan TJ (1998). Fungal endophytes: a continuum of interactions with host plants. Annu Rev Ecol Syst.

[CR59] Samson RA, Hoekstra ES, Frisvad JC (2004) Introduction to food- and airborne fungi (7th Ed.). Centraalbureau voor Schimmelcultures, Utrecht, The Netherlands. 389 p

[CR60] Schmid E, Oberwinkler F (1993). Mycorrhiza-like interaction between the achlorophyllous gametophyte *of Lycopodium clavatum* L. and its fungal endophyte studied by light and electron microscopy. New Phytol.

[CR61] Shannon CE (1948). A mathematical theory of communication. Bell Syst Tech J.

[CR62] Stone JK, Polishook JD, White JRJ, Mueller G, Bills GF, Foster MS (2004). Endophytic fungi. Biodiversity of fungi: Inventory and monitoring methods.

[CR63] Schulz B, Boyle C (2005). The endophytic continuum. Mycol Res.

[CR64] Sun X, Ding Q, Hyde KD, Guo LD (2012). Community structure and preference of endophytic fungi of three woody plants in a mixed forest. Fungal Ecol.

[CR65] Taylor TN, Hass H, Kerp H (1999). The oldest fossil ascomycetes. Nature.

[CR66] Taylor TN, Hass H, Kerp H, Krings M, Hanlin RT (2004). Perithecial ascomycetes from the 400 MaBP Rhynie Chert: an example of ancestral polymorphism. Mycologia.

[CR67] U’Ren JM, Lutzoni F, Miadlikowska J, Laetsch AD, Arnold AE (2012). Host- and geographic structure of endophytic and endolichenic fungal communities at a continental scale. Am J Bot.

[CR68] Wang B, Qiu Y-L (2006). Phylogenetic distribution and evolution of mycorrhizas in land plants. Mycorrhiza.

[CR69] Wang Y, Zeng QG, Zhang ZB, Yan RM, Wang LY, Zhu D (2011). Isolation and characterization of endophytic huperzine A-producing fungi from *Huperzia serrata*. J Ind Microbiol Biotechnol.

[CR70] Watanabe T (2002). Pictorial atlas of soil and seed fungi: morphologies of cultured fungi and key to species.

[CR71] Wearn JA, Sutton BC, Morley NJ, Gange AC (2012). Species and organ specificity of fungal endophytes in herbaceous grassland plants. J Ecol.

[CR72] White TJ, Bruns T, Lee S, Taylor J, Innis MA, Gelfland DH, Sninsky JJ, White TJ (1990). Amplification and direct sequencing of fungal ribosomal RNA genes for phylogenetics. PCR protocols: A guide to methods and applications.

[CR73] Whittier DP (1977). Gametophytes of *Lycopodium obscurum* grown in axenic culture. Can J Bot.

[CR74] Wilson D (1995). Endophytes—the evolution of the term, a clarification of its use and definition. Oikos.

[CR75] Xiang JG, Shan WG, Liang DE, Ying YM, Gan LS, Wang JW, Zhan ZJ (2013). N-gearing Furanone derivatives from an endophytic fungus in *Huperzia serrata*. Helv Chim Acta.

[CR76] Zhang ZB, Zeng QG, Yan RM, Wang Y, Zou ZR, Zhu D (2011). Endophytic fungus *Cladosporium cladosporioides* LF70 from *Huperzia serrata* produces Huperzine A. World J Microbiol Biotechnol.

[CR77] Zhou D, Hyde KD (2001). Host-specificity, host-exclusivity and host-recurrence in saprobic fungi. Mycol Res.

[CR78] Zhu D, Wang J, Zeng Q, Zhang Z, Yan R (2010). A novel endophytic Huperzine A–producing fungus, *Shiraia* sp. Slf14, isolated from *Huperzia serrata*. J Appl Microbiol.

