# Supplementary material for: The diversity of endophytic fungi in the above-ground tissue of two Lycopodium species in Poland
Source: Symbiosis. 2014 Sep 10;63(2):87–97. doi: 10.1007/s13199-014-0291-1 (PMC4174293; doi:10.1007/s13199-014-0291-1)
Supplement: Supplementary file 1 — (DOCX 25 kb) [file 13199_2014_291_MOESM1_ESM.docx]

Table S1. Coordinates of sites with date of collection, location, vegetation type, host plant and organ sampled.

| **Number** | **host plant** | **date** | **organ** | **coordinates** | | **location** | **Vegetation** |
| --- | --- | --- | --- | --- | --- | --- | --- |
| 1 | *L. clavatum* | 19.10.11 | shoot | 53,88856 | 23,44636 | lowland | mixed forests |
| 2 | *L. clavatum* | 19.10.11 | shoot | 53,87573 | 23,51799 | lowland | mixed forests |
|  | *L. clavatum* | 19.10.11 | strobilus | 53,87573 | 23,51799 | lowland | mixed forests |
| 3 | *L. clavatum* | 10.08.11 | shoot | 50,99220 | 19,09023 | highland | mixed forests |
| 4 | *L. clavatum* | 03.09.11 | strobilus | 51,02776 | 19,09023 | highland | mixed forests |
| 5 | *L. clavatum* | 03.09.11 | strobilus | 51,01433 | 19,09023 | highland | mixed forests |
| 6 | *L. clavatum* | 12.07.11 | strobilus | 51,13985 | 18,74853 | high land | pine forest |
| 7 | *L. clavatum* | 12.07.11 | strobilus | 51,13863 | 18,74898 | high land | pine forest |
| 8 | *L. clavatum* | 16.07.11 | strobilus | 50,33455 | 19,47132 | highland | pine forest |
| 9 | *L. clavatum* | 16.07.11 | strobilus | 50,32625 | 19,46972 | highland | pine forest |
| 10 | *L. clavatum* | 17.07.11 | strobilus | 50,33132 | 19,48460 | highland | pine forest |
| 11 | *L. clavatum* | 02.10.11 | shoot | 50,99596 | 19,64848 | highland | pine forest |
| 12 | *L. clavatum* | 02.10.11 | shoot | 50,98968 | 19,66256 | highland | pine forest |
| 13 | *L. clavatum* | 02.10.11 | strobilus | 50,99596 | 19,64848 | highland | pine forest |
| 14 | *L. clavatum* | 02.10.11 | strobilus | 50,98968 | 19,66256 | highland | pine forest |
| 15 | *L. clavatum* | 02.10.11 | strobilus | 50,63065 | 18,67955 | highland | pine forest |
| 16 | *L. clavatum* | 02.08.11 | strobilus | 50,75716 | 18,85822 | highland | acidophilic oak forests |
| 17 | *L. clavatum* | 02.08.11 | strobilus | 50,76783 | 18,86705 | highland | acidophilic oak forests |
| 18 | *L. clavatum* | 06.08.11 | strobilus | 50,35582 | 19,60240 | highland | pine forest |
| 19 | *L. clavatum* | 25.09.11 | shoot | 49,57605 | 18,97302 | mountain | mountain spruce forests |
| 20 | *L. clavatum* | 25.09.11 | shoot | 49,57676 | 18,97076 | mountain | mountain spruce forests |
| 21 | *L. clavatum* | 25.09.11 | shoot | 49,57742 | 18,96780 | mountain | mountain spruce forests |
| 22 | *L. clavatum* | 25.09.11 | strobilus | 49,57665 | 18,97188 | mountain | mountain spruce forests |
|  | *L. clavatum* | 25.09.11 | shoot | 49,57665 | 18,97188 | mountain | mountain spruce forests |
| 23 | *L. clavatum* | 25.09.11 | strobilus | 49,57676 | 18,97076 | mountain | mountain spruce forests |
| 24 | *L. clavatum* | 25.09.11 | shoot | 49,63663 | 19,00105 | mountain | mountain spruce forests |
| 25 | *L. clavatum* | 05.07.11 | strobilus | 50,54669 | 19,47600 | highland | pine forest |
| 26 | *L. clavatum* | 09.08.11 | shoot | 50,69115 | 19,22263 | highland | pine bog forest |
| 27 | *L. clavatum* | 09.08.11 | shoot | 50,65030 | 19,18702 | highland | pine forest |
| 28 | *L. annotinum* | 19.10.11 | strobilus | 53,87573 | 23,51799 | lowland | mixed forest |
|  | *L. annotinum* | 19.10.11 | shoot | 53,87573 | 23,51799 | lowland | mixed forests |
| 29 | *L. annotinum* | 19.10.11 | strobilus | 53,89610 | 23,50030 | lowland | pine bog forest |
|  | *L. annotinum* | 19.10.11 | shoot | 53,89610 | 23,50030 | lowland | pine bog forest |
| 30 | *L. annotinum* | 19.10.11 | shoot | 53,89500 | 23,51390 | lowland | mixed forests |
| 31 | *L. annotinum* | 19.10.11 | strobilus | 53,88890 | 23,41194 | lowland | pine bog forest |
|  | *L. annotnum* | 19.10.11 | shoot | 53,88890 | 23,41194 | lowland | pine bog forest |
| 32 | *L. annotinum* | 10.08.11 | shoot | 50,99046 | 19,09053 | highland | mixed forests |
|  | *L. annotinum* | 10.08.11 | strobilus | 50,99046 | 19,09053 | highland | mixed forests |
| 33 | *L. annotinum* | 10.08.11 | shoot | 50,98846 | 19,10136 | highland | mixed forests |
|  | *L. annotinum* | 10.08.11 | strobilus | 50,98846 | 19,10136 | highland | mixed forests |
| 34 | *L. annotinum* | 10.08.11 | shoot | 50,98618 | 19,09768 | highland | mixed forests |
|  | *L. annotinum* | 10.08.11 | strobilus | 50,98618 | 19,09768 | highland | mixed forests |
| 35 | *L. annotinum* | 10.08.11 | strobilus | 50,99132 | 19,09155 | highland | mixed forests |
|  | *L. annotinum* | 10.08.11 | shoot | 50,99132 | 19,09155 | highland | mixed forests |
| 36 | *L. annotinum* | 09.09.11 | shoot | 51,13498 | 18,76648 | highland | pine forest |
|  | *L. annotnium* | 09.09.11 | strobilus | 51,13498 | 18,76648 | highland | pine forest |
| 37 | *L. annotinum* | 09.09.11 | shoot | 51,13478 | 18,74175 | highland | pine forest |
|  | *L. annotinum* | 09.09.11 | strobilus | 51,13478 | 18,74175 | highland | pine forest |
| 38 | *L. annotinum* | 18.07.11 | strobilus | 50,34205 | 19,68850 | highland | pine forest |
| 39 | *L. annotinum* | 18.07.11 | strobilus | 50,23845 | 19,53452 | highland | pine forest |
| 40 | *L. annotinum* | 02.10.11 | shoot | 50,99658 | 19,64888 | highland | pine forest |
| 41 | *L. annotinum* | 02.10.11 | shoot | 50,60582 | 18,69615 | highland | pine forest |
|  | *L. annotinum* | 02.10.11 | strobilus | 50,60582 | 18,69615 | highland | pine forest |
| 42 | *L. annotinum* | 02.10.11 | shoot | 50,63038 | 18,67328 | highland | pine forest |
| 43 | *L. annotinum* | 02.10.11 | strobilus | 50,63093 | 18,67386 | highland | pine forest |
| 44 | *L. annotinum* | 02.08.11 | strobilus | 50,79485 | 18,67898 | highland | acidophilic oak forests |
| 45 | *L. annotinum* | 02.08.11 | strobilus | 50,77120 | 18,88006 | highland | acidophilic oak forests |
| 46 | *L. annotinum* | 20.07.11 | strobilus | 50,71523 | 19,50410 | highland | acidophilic beech forests |
| 47 | *L. annotinum* | 25.07.11 | strobilus | 50,32063 | 19,50113 | highland | pine forest |
| 48 | *L. annotinum* | 27.07.11 | shoot | 50,33242 | 19,51356 | highland | pine bog forest |
| 49 | *L. annotinum* | 08.08.11 | shoot | 50,52505 | 19,86525 | highland | pine bog forest |
|  | *L. annotinum* | 08.08.11 | strobilus | 50,52505 | 19,86525 | highland | pine bog forest |
| 50 | *L. annotinum* | 25.09.11 | shoot | 49,62332 | 19,00746 | mountain | mountain spruce forests |
|  | *L. annotinum* | 25.09.11 | strobilus | 49,62332 | 19,00746 | mountain | mountain spruce forests |
| 51 | *L. annotinum* | 25.09.11 | strobilus | 49,61118 | 18,99642 | mountain | mountain spruce forests |
| 52 | *L. annotinum* | 05.07.11 | strobilus | 50,64545 | 19,10052 | highland | pine forest |
| 53 | *L. annotinum* | 27.07.11 | strobilus | 50,64706 | 19,08918 | highland | pine bog forest |
| 54 | *L. annotinum* | 04.09.11 | shoot | 50,73558 | 19,47873 | highland | pine bog forest |
| 55 | *L. annotinum* | 07.07.11 | strobilus | 50,75966 | 19,43286 | highland | pine bog forest |
| 56 | *L. annotinum* | 05.07.11 | strobilus | 50,54669 | 19,47600 | highland | pine forest |
|  | *L. annotinum* | 05.07.11 | shoot | 50,54669 | 19,47600 | highland | pine forest |
| 57 | *L. annotinum* | 05.07.11 | strobilus | 50,54630 | 19,47560 | highland | pine forest |
| 58 | *L. annotinum* | 08.08.11 | strobilus | 50,70372 | 19,21765 | highland | pine bog forest |
| 59 | *L. annotinum* | 09.08.11 | strobilus | 50,64962 | 19,18585 | highland | pine forest |
| 60 | *L. annotinum* | 09.08.11 | strobilus | 50,69115 | 19,22263 | highland | pine forest |
| 61 | *L. annotinum* | 09.08.11 | shoot | 50,70372 | 19,21765 | highland | pine bog forest |
| 62 | *L. annotinum* | 09.08.11 | shoot | 50,64962 | 19,18585 | highland | pine bog forest |
| 63 | *L. annotinum* | 09.08.11 | shoot | 50,69452 | 19,20755 | highland | pine bog forest |
